# PCGEM1 promotes proliferation, migration and invasion in prostate cancer by sponging miR-506 to upregulate TRIAP1

**DOI:** 10.1186/s12894-022-00969-x

**Published:** 2022-02-02

**Authors:** He Liu, Xin He, Tianjiao Li, Yi Qu, Lina Xu, Yingnan Hou, Yao Fu, Hongzhi Wang

**Affiliations:** 1 Department of Urology, Heilongjiang Red Cross Sengong General Hospital, 32 Hexing Rd, Xiangfang District, Harbin, 150001 Heilongjiang Province China; 2 Department of General Surgery, Heilongjiang Red Cross Sengong General Hospital, Harbin, 150001 Heilongjiang Province China

**Keywords:** Prostate cancer, Circular RNAs, PCGEM1, miR‑506‑3p, TRIAP1

## Abstract

**Background:**

The important role of long noncoding RNAs (lncRNAs) in cancer has been demonstrated in many studies. Prostate cancer gene expression marker 1 (PCGEM1) is a lncRNA specifically expressed within the prostate and overexpressed in many cancer cells. Numerous studies have shown that PCGEM1 promotes cell proliferation, invasion and migration. However, the specific mechanism of PCGEM1 within prostate cancer (PCa) has not been elucidated. MicroRNA-506-3p (miR-506-3p) is a noncoding RNA, and studies have indicated that miR-506-3p is downregulated in prostate cancer cell lines and functions as a tumor suppressor.

**Methods:**

The TCGA (GEPIA) database (http://gepia.cancer-pku.cn/) was employed to measure PCGEM1 levels in PCa. Quantitative real-time polymerase chain reaction (qRT-PCR) was performed to determine the PCGEM1 gene level. CCK-8 (Cell Counting Kit-8) and colony formation assays were used to detect cell proliferation, and Transwell assays were applied to assess cell invasion and migration. The interacting ability of miR-506-3p with PCGEM1 or TRIAP1 was validated through a dual-luciferase reporter assay. TRIAP1 protein expression was detected by Western blotting.

**Results:**

PCGEM1 expression was increased in PCa tissues and cells. In PCa tissues, High PCGEM1 expression was associated with high Gleason score, distant metastasis and extracapsular extension. In addition, PCGEM1 knockdown inhibited PCa cell (C4-2B and PC-3) proliferation, invasion and migration. miR-506-3p may interact with PCGEM1 or TRIAP1, and the suppressive effect of PCGEM1 knockdown was reversed when TRIAP1 or a miR-506-3p inhibitor was cotransfected.

**Conclusion:**

PCGEM1 expression increased in PCa cells and tissues, enhancing PCa cell proliferation, migration and invasion by sponging miR-506 to upregulate TRIAP1.

**Supplementary Information:**

The online version contains supplementary material available at 10.1186/s12894-022-00969-x.

## Background

Prostate cancer (PCa) is a common male cancer and also the second most common cause of cancer-related mortality in the USA [1]. Prostatic epithelial cells specifically express serum prostate-specific antigen (PSA). Therefore, the PSA test is utilized for the early detection of PCa and as a biomarker for disease diagnosis and efficacy monitoring [2]. Although PSA can effectively identify PCa, there are often some false positives. Therefore, additional PCa-specific molecular markers should be identified to improve the diagnosis and prognosis of PCa. Various research methods have been used to reveal the mechanism of PCa and identify prostate-specific genes for the early detection and diagnosis of PCa.

Long noncoding RNAs (lncRNAs) are noncoding RNAs over 200 nucleotides in length [3]. Aberrant levels of lncRNAs have been detected in many cancers and play important roles in modulating cell proliferation, apoptosis, metastasis and invasion. However, the detailed mechanism is still unclear. LncRNAs have been shown to modulate downstream target gene levels by interacting with microRNAs (miRNAs) [3, 4]. Prostate cancer gene expression marker 1 (PCGEM1) is a well-recognized early carcinogenic lncRNA in PCa [5] that is markedly increased in PCa tissue relative to noncarcinoma tissues, and increased PCGEM1 expression is associated with a family history of PCa [6]. PCGEM1 regulates the proliferation of castration-recurrent prostate cancer (CRPC) by binding to the androgen receptor and may be adopted as the necessary CRPC component [7, 8]. Although there have been many studies on PCGEM1 in the past 15 years, the role of PCGEM1 in transcriptional regulation is not completely clear.

MicroRNAs can regulate gene expression through the 3’-untranslated region (3’-UTR), and evidence shows that miRNAs play a crucial role in the initiation and progression of human cancers, including PCa [9, 10]. In addition, aberrant expression of miR-506-3p inhibits cancer cell growth, migration, and invasion through inactivation of the Wnt/β-catenin signaling pathway [11]. In the glioma U251 cell line, miR-506 upregulation inhibited the expression of proliferation-, apoptosis-, migration- and invasion-related proteins [12].

The tumorigenesis is a process involving multiple stages and factors. Apoptosis is a basic biological process and plays a key role in individual growth, development and other life stages. TP53-regulated inhibitor of apoptosis 1 (TRIAP1) is a new type of apoptosis inhibitor, TRIAP1 has been shown to be upregulated in different human malignancies, and associated with resistance of apoptosis. TRIAP1 was significantly in penile carcinoma (PeCa), TRIAP1 expression was significantly related with the histological grade and the local recurrence rate [13]. Irradiation notably increased the levels of TRIAP1 in non-small cell lung cancer (NSCLC) cells, TRIAP1 was a key factor in NSCLC radioresistance by maintaining redox homeostasis [14].

The present work was conducted to explore the mechanisms of PCGEM1 in regulating PCa cell proliferation, metastasis and invasion. Such knowledge may contribute to the development of targeted therapy and improve the prognosis of PCa.

## Materials and methods

### GEPIA analysis

TCGA (GEPIA) (http://gepia.cancer-pku.cn/index.html) was adopted for analyzing PCGEM1 mRNA levels in PCa. The |Log_2_FC| Cutoff and *P*-value Cutoff used default value as 1 and 0.001 [15].

### Samples collection

50 PCa tissue samples (PCa), together with the matched non-carcinoma tissue samples (Normal) without any treatment before the surgery were collected from Department of Urology, Heilongjiang Red Cross Sengong General Hospital. Our study protocol gained approval from the Ethics Committee. Each patient provided the informed consent for participation.

### Cell culture

The human PCa cells (PC-3, LNPCa, Du-145, C4-2B), along with the normal prostate cell line (RWPE1) were provided by Chinese Academy of Sciences Cell Bank. In accordance with ATCC website, each cell line was cultured within the base medium which contained 10% fetal bovine serum (FBS), followed by incubation under 37℃ and 5% CO_2_ conditions [5].

### Quantitative real‑time polymerase chain reaction (qRT‑PCR)

TRIzol was used to extract total RNA, and Transcriptor cDNA Synth Kit (Roche, USA) was utilized to prepare cDNA. Afterwards, the FS Universal SYBR Green Master (Roche, USA) was used to measure target gene expression level, which was calculated using 2^−ΔΔCt^. qRT-PCR was performed on LightCycler 480 (Roche), with GAPDH and U6 being the internal reference genes [16]. The primers were as follows: miR-506-3p: F: 5′-TAAGGCACCCTTCTGAGTAGA-3′ and R: 5′-GCGAGCACAGAATTAATACGAC-3′; U6 snRNA: F: 5′-TGACACGCAAATTCGTGAAGCGTTC-3′ and R: 5′-CCAGTCTCAGGGTCCGAGGTATTC-3′; GAPDH: F: 5′-GGTGTGAACCATGAGAAGTATGA-3′ and R: 5′-GAGTCCTTCCACGATACCAAAG-3′; PCGEM1: F: 5′-CTGTGTCTGCAACTTCCTCTAA-3′ and R: 5′-TCCCAGTGCATCTCGTAGTA-3′. TRIAP1: F: 5′-AGGATTTCGCAAGTCCAGAA-3′ and R: 5′-GCTGATTCCACCCAAGTAT-3′.

### Cell transfection

The PCGEM1-targeting lentiviral short hairpin RNA (shRNA, si-PCGEM1#1/2), TRIAP1 overexpression plasmid, miR-506-3p inhibitor, miR-506-3p mimic, miR-NC, together with negative control (vector) were provided by GENECHEM (Shanghai, China). Plasmids were transfected in C4-2B and PC-3 cells using Lipofectamine 2000 (Invitrogen, USA).

### Cell proliferation assay

C4-2B and PC-3 cell proliferation was analyzed through Cell Counting Kit-8 (CCK-8) and colony formation assays. For CCK8 assay, we counted the C4-2B and PC-3 cell numbers and inoculated them into 96-well plates. 24 h later, we added 10μL CCK8 solution into each 96-well plate for 4 h before the measurement of OD value at 450 nm [17]. For colony formation assays, The C4-2B and PC-3 cell numbers were determined, then cells were inoculated to the 6-well plates for colony formation assay. After 2 weeks of incubation, the above two cell lines were subjected to 4% paraformaldehyde fixation, followed by 1% crystal violet staining. Finally, the microscope (Olympus, Japan) was used to capture images and the Image J software (NIH, USA) was adopted to count the colonies [18].

### Transwell assay

Transwell assay was utilized for detecting cell invasion, as well as migration capacities of C4-2B and PC-2 cells with or without Matrigel (BD Biosciences, USA). Firstly, the C4-2B and PC-3 cell numbers were determined, then cells were inoculated to upper chamber coated with serum-free medium, whereas complete growth medium was put to lower chamber. 24 h later, the above cells were subjected to 4% paraformaldehyde fixation, followed by 1% crystal violet staining. Finally, the microscope (Olympus, Japan) was used to capture images and the Image J software (NIH, USA) was adopted to count the number of invasion and migration cells.

### Subcellular fractionation and localization

Cytoplasmic or nuclear RNA purification kit (Norgen BioTek, Canada) was utilized for extracting cytoplasmic or nuclear RNA, respectively. Thereafter, the cytoplasmic and nuclear PCGEM1 levels were determined through qRT-PCR. At last, qRT-PCR results were normalized based on GAPDH (cytoplasmic control) and U6 (nuclear control).

### Dual‑luciferase reporter assay

PCGEM1 and TRIAP1 sequences in the presence (WT) or absence (MUT) of the predicted biding sites of miR-506-3p were prepared by Invitrogen (Thermo fisher Scientific, USA) and inserted into psiCHECK-2™ vector (Promega, USA). Cells with co-transfection of reporter vector and miR-506-3p mimic or miR-NC were used to test luciferase activity by a Dual-Luciferase® Reporter Assay System (Promega, USA) [19].

### Biotin‑labeled pull‑down assay

Biotin-labeled miR-506-3p (miR-506-3p-probe) and negative control (NC-probe) were purchased from Sangon Biotech (Shanghai, China). The above probes were transfected into C4-2B and PC-2 cells, 48 h later, the cells were lysed and incubated with magnetic beads at 4 °C for 3 h. The enrichment of PCGEM1 were detected by qRT-PCR [17].

### RNA immunoprecipitation (RIP) assay

C4-2B and PC-2 cells was lysed using RIP lysis buffer (Millipore, USA). Then, cell lysate was incubated with magnetic beads (Millipore) bound to argonaute2 antibody (Anti-Ago2) or immunoglobulin G (IgG) antibody (Anti-IgG). The part of cell lysates not incubated with magnetic beads was used as control (Input). The coprecipitated RNAs were purified and detected by qRT-PCR [17].

### Western blotting (WB)

The RIPA lysis buffer (Beyotime, China) supplemented with PMSF (Beyotime, China) was used to extract total protein following specific protocols. The BCA kit (Beyotime, China) was adopted to quantify the protein. Then, equivalent proteins were isolated through SDS-PAGE, followed by transfer to the nitrocellulose membrane (Millipore, USA). Then, 5% fat-free milk was utilized to block membranes for 1 h under ambient temperature, and anti-TRIAP1 (1:1000, Invitrogen, USA) and anti-GAPDH (1:10,000, Abcam, USA) primary antibodies were adopted for incubating cells overnight under 4 °C. Thereafter, secondary antibody (1:10,000, Abcam, USA) was added to incubate for another 1 h under ambient temperature. After washing, the protein bands were detected by BeyoECL plus kit (Beyotime, China). Relative protein expression was analyzed using Image J software (NIH, USA).

### In vivo tumorigenesis model

4-week-old female BALB/c nude mice were purched from Charles river (Beijing, China), and were randomly divided into two groups. PC-3 cells transfected with si-NC and si-PCGEM1#1 were injected into mice subcutaneously (3 × 10^6^ cells), then the tumor volume were measured every 7 days, tumor volume was calculated as 1/2 × (length) × (width)^2^. After 28 days, mice were sacrificed followed by tumor weight measurement. Our study protocol gained approval from the Animal Care and Use Committee.

### Statistical analysis

Significance in groups was compared by student’s t-test, one-way or repeated measures *ANOVA* analysis of variance in SPSS22.0 (SPSS Inc., USA). All results were presented in the manner of the mean ± SD. *P* < 0.05 was considered statistically significant.

## Results

### PCGEM1 is overexpressed in PCa cells and tissues

**Fig. 1:**
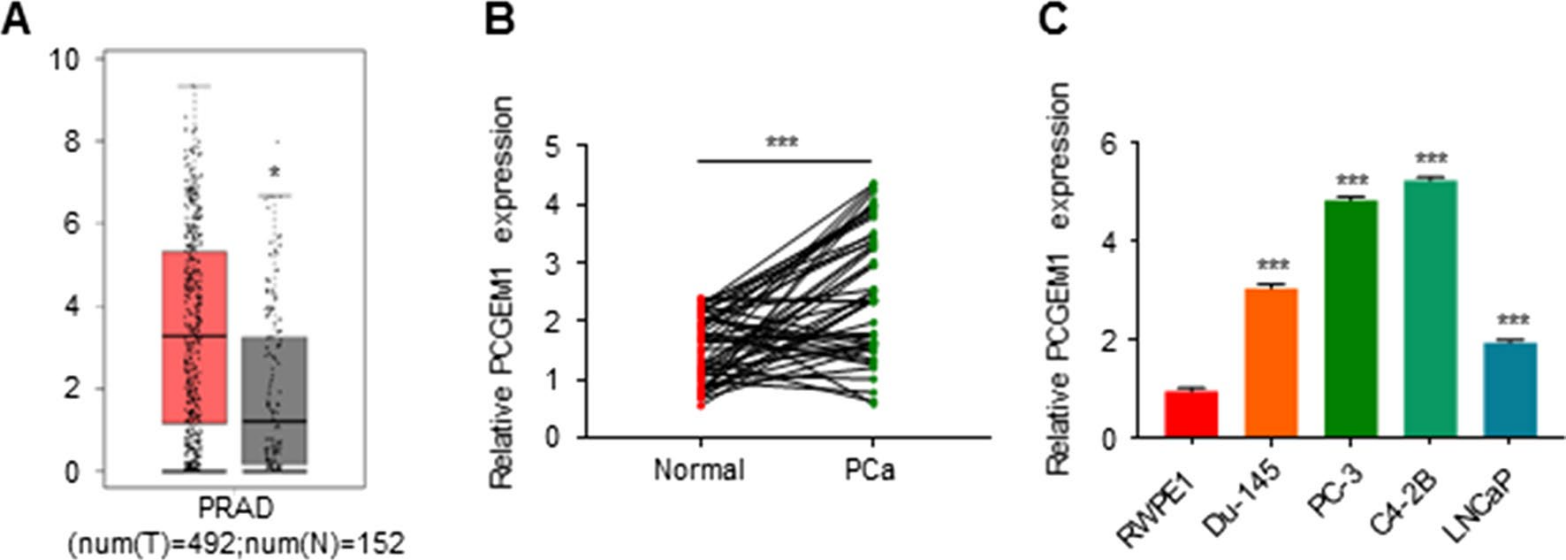
PCGEM1 levels within PCa tissues and cell lines. **A** Box plots showing increased PCGEM1 expression within PCa tissues compared with normal tissues. **B** PCGEM1 mRNA expression in 50 PCa cases was measured by qRT-PCR. **C** PCGEM1 mRNA expression in PCa cells (LNCaP, PC-3, C4-2B and Du-145) and noncarcinoma prostate RWPE1 cells was measured by qRT-PCR. *P < 0.05, ***P < 0.001

The PCGEM1 level in PCa was analyzed via TCGA (GEPIA) using the cutoff threshold of *P* < 0.001 and log2 |fold change|> 1. The PCGEM1 level was increased in PCa samples relative to normal samples (Fig. 1A). Therefore, 50 PCa tissue samples together with the matched noncarcinoma samples were selected to validate the PCGEM1 level. Statistical analysis showed that PCGEM1 levels in PCa samples were increased relative to those in normal samples (Fig. 1B). 50 PCa patients were divided into two groups: high PCGEM1 expression and low PCGEM1 expression groups using the median value. The expression of PCGEM1 was not related to age, PSA and TNM stage. Interestingly, high PCGEM1 expression was associated with high Gleason score, distant metastasis and extracapsular extension significantly (Table 1, Additional file 1: Fig. S1A). Moreover, we found that PCGEM1 levels in four PCa cell lines (especially PC-3 and C4-2B) were higher than those in RWPE1 cells (Fig. 1C). All data revealed that PCGEM1 played a vital role in PCa. As a result, PCGEM1 was chosen for further analysis.

**Table 1:** Clinicopathological characteristics and PCGEM1 expression of 50 prostate cancer patients

Clinicopathological characteristics	Number	Low PCGEM1 expression (n = 25)	High PCGEM1 expression (n = 25)	*P* value
Age				0.5637
≥ 60	20	9	11	
< 60	30	16	14	
Gleason score				0.0109
≤ 8	25	17	8	
> 8	25	8	17	
PSA				0.6063
< 10 ng/mL	16	9	7	
≥ 10 ng/mL	34	16	18	
TNM stage				0.0771
I + II	32	19	13	
III + IV	18	6	12	
Distant metastasis				0.0235
M0	26	17	9	
M1	24	8	16	
Extracapsular extension			0.0366	
Negative	33	20	13	
Positive	17	5	12	

### Knockdown of PCGEM1 inhibited PCa proliferation, invasion and migration

**Fig. 2:**
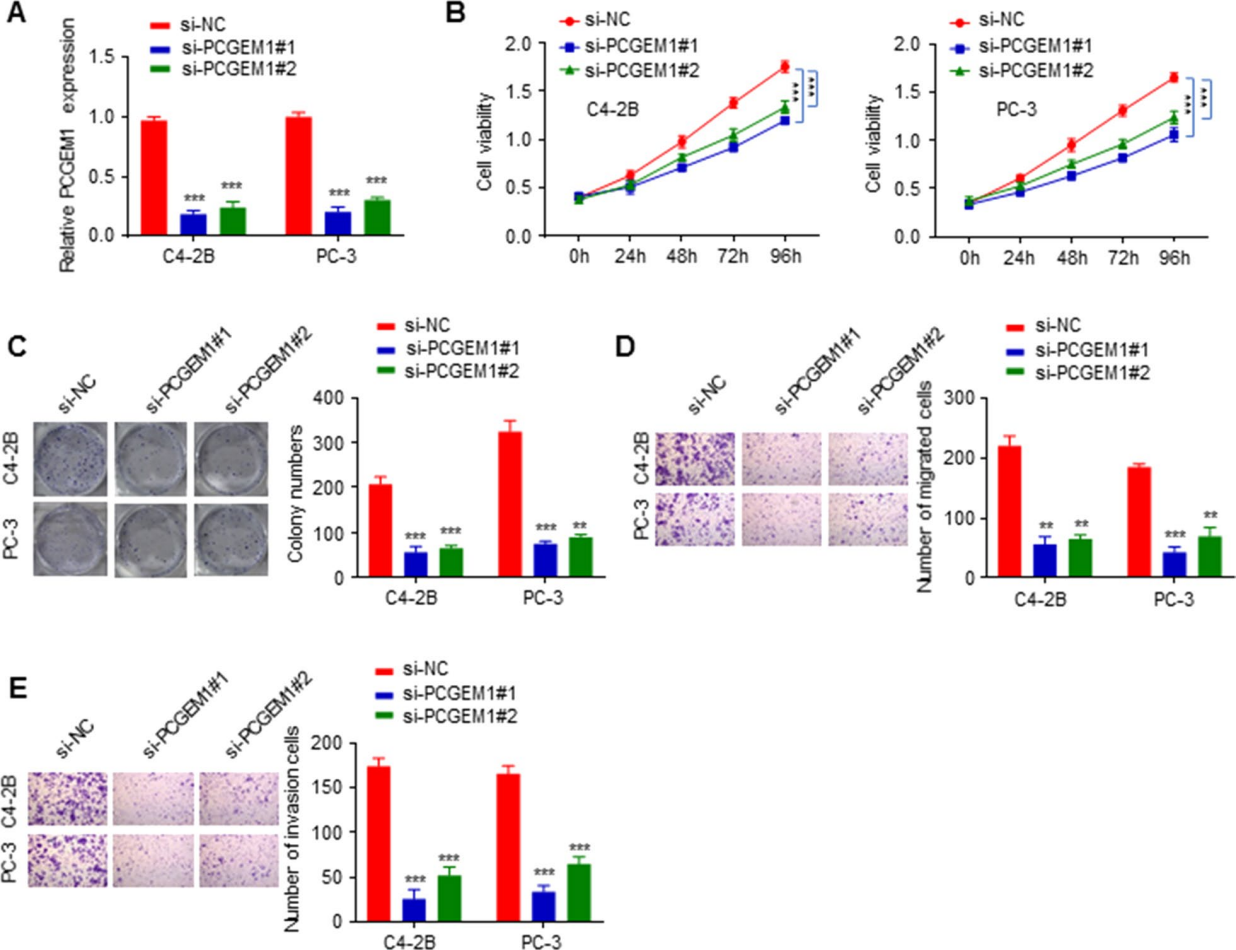
PCGEM1 knockdown suppressed PC-3 and C4-2B cell proliferation, invasion and migration. **A** Transfection efficiency measured by qRT-PCR. **B**, **C** C4-2B and PC-3 cell proliferation was measured by CCK-8 assay and colony formation assay. **D**, **E** Migration and invasion were measured by Transwell assay. **P < 0.01, ***P < 0.001

To explore the function of PCGEM1 in PCa cells, si-PCGEM1#1 or si-PCGEM1#2 was transfected into PC-3 and C4-2B cell lines. qRT-PCR results showed that si-PCGEM1#1 and si-PCGEM1#2 remarkably reduced the expression of PCGEM1 (Fig. 2A). CCK-8 and colony formation assay results showed that knockdown of PCGEM1 markedly decreased the viability and colony numbers of PC-3 and C4-2B cells (Fig. 2B, C). Additionally, the number of migrated and invaded cells was inhibited after PCGEM1 knockdown (Fig. 2D, E). These data showed that PCGEM1 might be associated with oncogenesis in PCa cells, and that PCGEM1 knockdown restrained PCa cell proliferation, migration and invasion.

### PCGEM1 directly sponged miR-506-3p

**Fig. 3:**
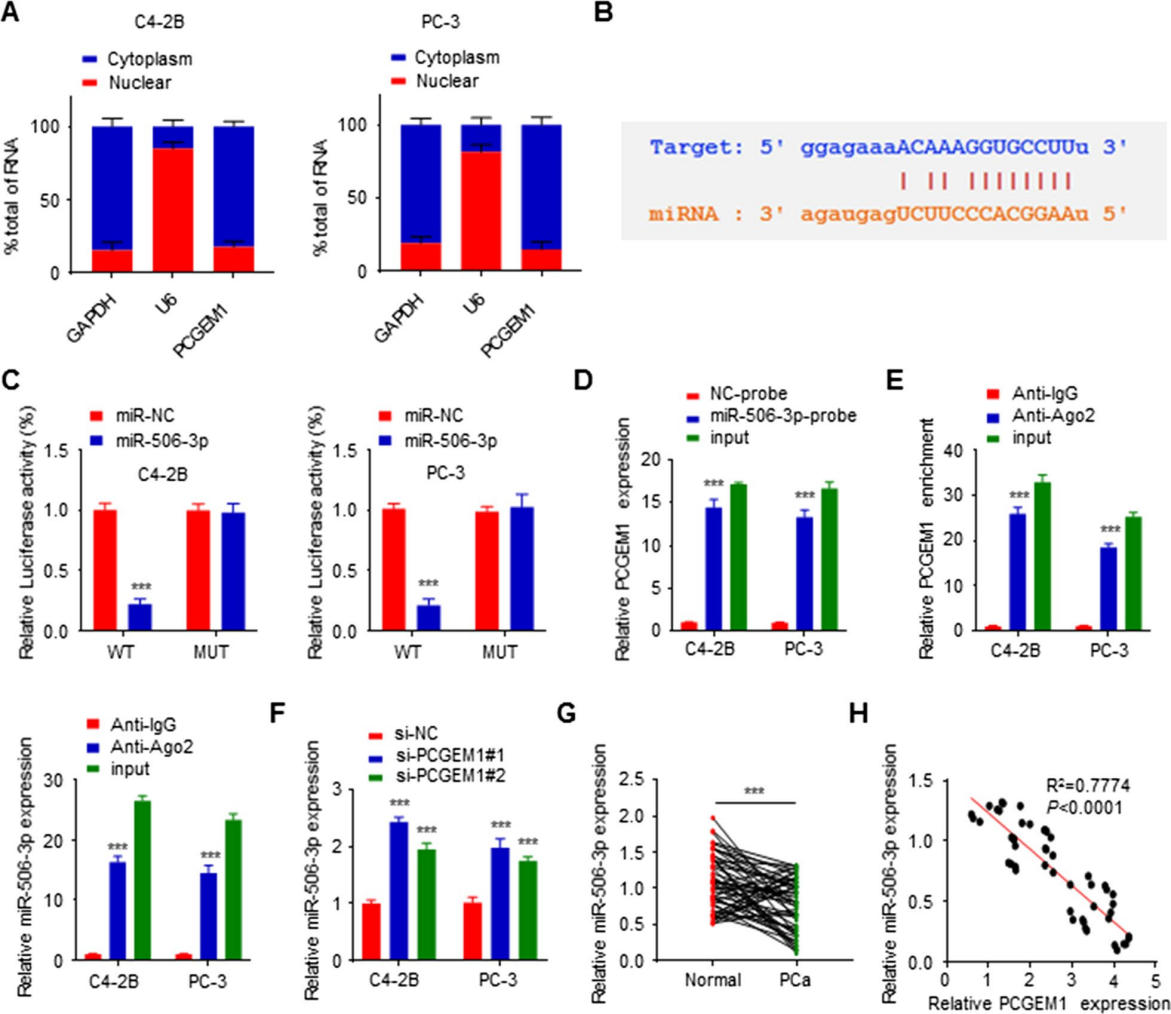
PCGEM1 directly sponged miR-506-3p. **A** The subcellular localization and fractionation of PCGEM1 within PC-3 and C4-2B cells were determined through qRT-PCR, with GAPDH (cytoplasmic) and U6 (nuclear) as internal references. **B** Binding sites in PCGEM1 versus miR-506-3p. **C**, **D** Binding sites in PC-3 and C4-2B cells were investigated by dual-luciferase assay and biotin-labeled pull-down assay. **E** RIP assay was performed to determine the enrichment of PCGEM1 and miR-506-3p. **F** MiR-506-3p mRNA expression in PC-3 and C4-2B cells with PCGEM1 knockdown detected by qRT-PCR. **G** miR-506-3p levels in 50 PCa cases were measured by qRT-PCR. **H** The relationship of miR-506-3p with PCGEM1 levels was measured through Pearson correlation analysis. ***P < 0.001

Through analyzing the subcellular localization of PCGEM1, we found that PCGEM1 was mainly localized within the cytoplasm of PC-3 and C4-2B cells (Fig. 3A), indicating that PCGEM1 might regulate posttranscriptional pathways by interacting with miR-506-3p. Therefore, the ENCORI was employed to predict the binding sites in PCGEM1 and miR-506-3p (Fig. 3B). The prediction result was validated through a luciferase reporter assay, suggesting that miR-506-3p overexpression markedly decreases luciferase activities in PCGEM1-WT cells compared to PCGEM1-MUT cells (Fig. 3C). A biotin-labeled pull-down assay indicated that PCGEM1 could be pulled down by the miR-506-3p probe compared to the NC probe (Fig. 3D). Furthermore, a RIP assay also confirmed that PCGEM1 and miR-506-3p could be enriched using anti-Ago2 compared to anti-IgG antibodies (Fig. 3E). In addition, miR-506-3p levels increased in PC-3 and C4-2B cells after PCGEM1 knockdown (Fig. 3F). These data demonstrated that PCGEM1 directly sponged miR-506-3p. Furthermore, the miR-506-3p levels were measured in 50 PCa samples and matched noncarcinoma samples, and the miR-506-3p expression was decreased in PCa tissues relative to noncarcinoma tissue samples (Fig. 3G, Additional file 2: Fig. S2A). Furthermore, correlation analysis was also performed, and a negative correlation of the PCGEM1 level with miR-506-3p within PCa tissues was observed (Fig. 3H). These results confirmed the interaction between PCGEM1 and miR-506-3p.

### miR-506-3p targeted TRIAP1

**Fig. 4:**
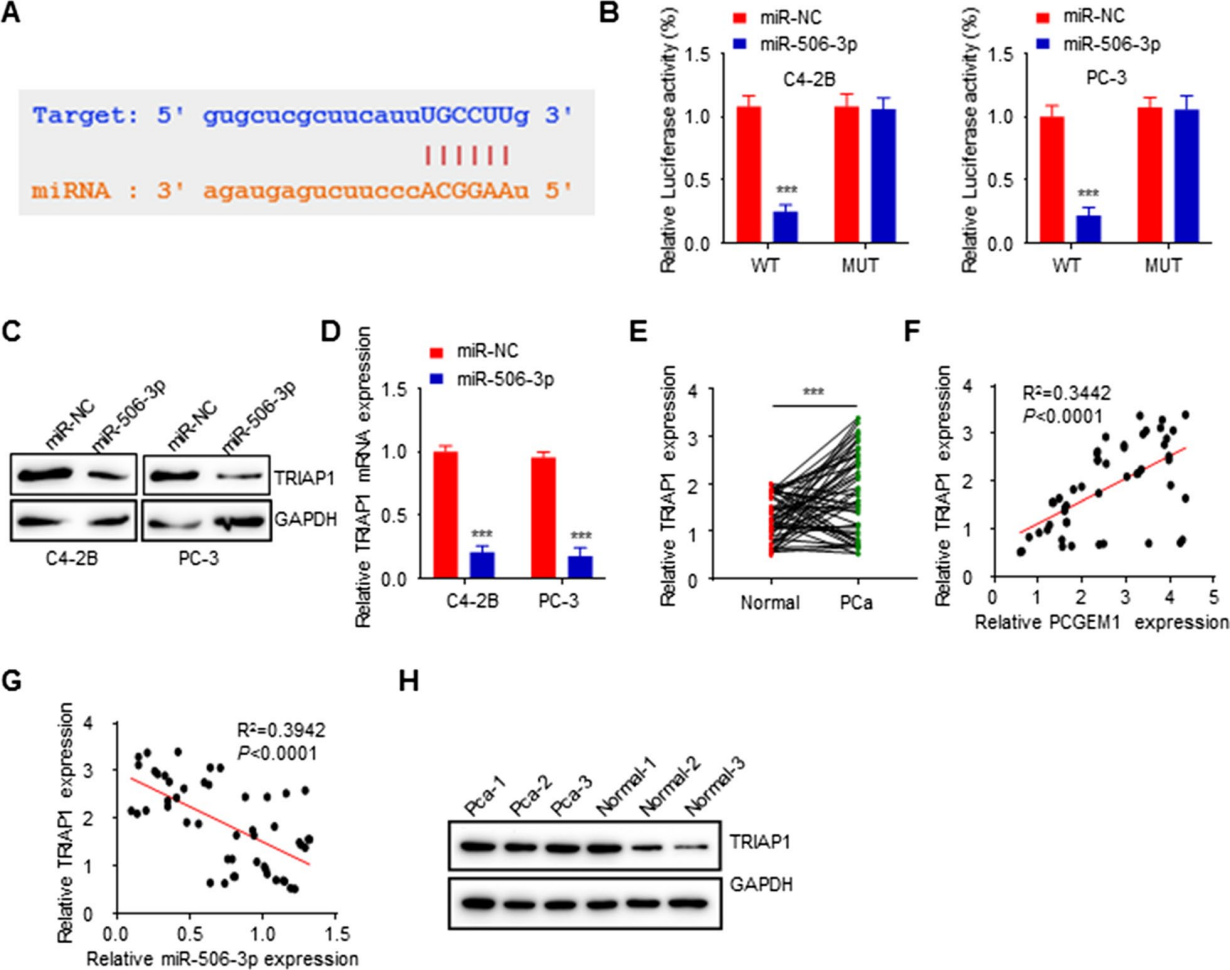
MiR‑506‑3p may target TRIAP1. **A** MiR-506-3p binding sites within TRIAP1 3’UTR. **B** Associations of miR-506-3p with the TRIAP1 3’UTR in PC-3 and C4-2B cells were shown by dual-luciferase assay. **C**, **D** miR-506-3p mimic effects on TRIAP1 protein and mRNA expressions in PC-3 and C4-2B cells were determined through Western blotting and qRT-PCR. **E** TRIAP1 mRNA expression in 50 PCa cases was measured by qRT-PCR. **F**, **G** The relationship between TRIAP1 and PCGEM1 or miR‑506‑3p was determined through Pearson correlation analysis. **H** The protein expression of TRIAP1 in 3 paired cases was measured by WB. ***P < 0.001

Next, we predicted the genes targeted by miR-506-3p via the ENCORI. According to the literature and our previous studies, we selected the TRIAP1 gene for further study, and miR-506-3p binding sites in the TRIAP1 3′UTR are shown in Fig. 4A. A luciferase reporter assay was performed to verify the binding sites and showed that miR-506-3p overexpression markedly decreased luciferase activities in TRIAP1-WT cells but not TRIAP1-MUT cells (Fig. 4B). Moreover, miR-506-3p overexpression inhibited TRIAP1 protein and mRNAs level in PC-3 and C4-2B cells (Fig. 4C, D). Consistently, the TRIAP1 mRNA level was upregulated in PCa tissues relative to matched noncarcinoma tissues (Fig. 4E, Additional file 2: Fig. S2B, C). What’s more, the protein expression levels of RIAP1 were higher in prostate cancer patients with high Gleason score (Additional file 1: Fig. S1B). Notably, the TRIAP1 level was positively correlated with the PCGEM1 level, whereas negatively correlated with miR-506-3p level in PCa tissues (Fig. 4F, G). The protein level of TRIAP1 was upregulated in PCa tissues relative to matched noncarcinoma tissues (Fig. 4H). These findings verified that TRIAP1 was the miR-506-3p target.

### PCGEM1 regulated the biological function of PCa cells by interacting with miR‑506‑3p to upregulate TRIAP1

**Fig. 5:**
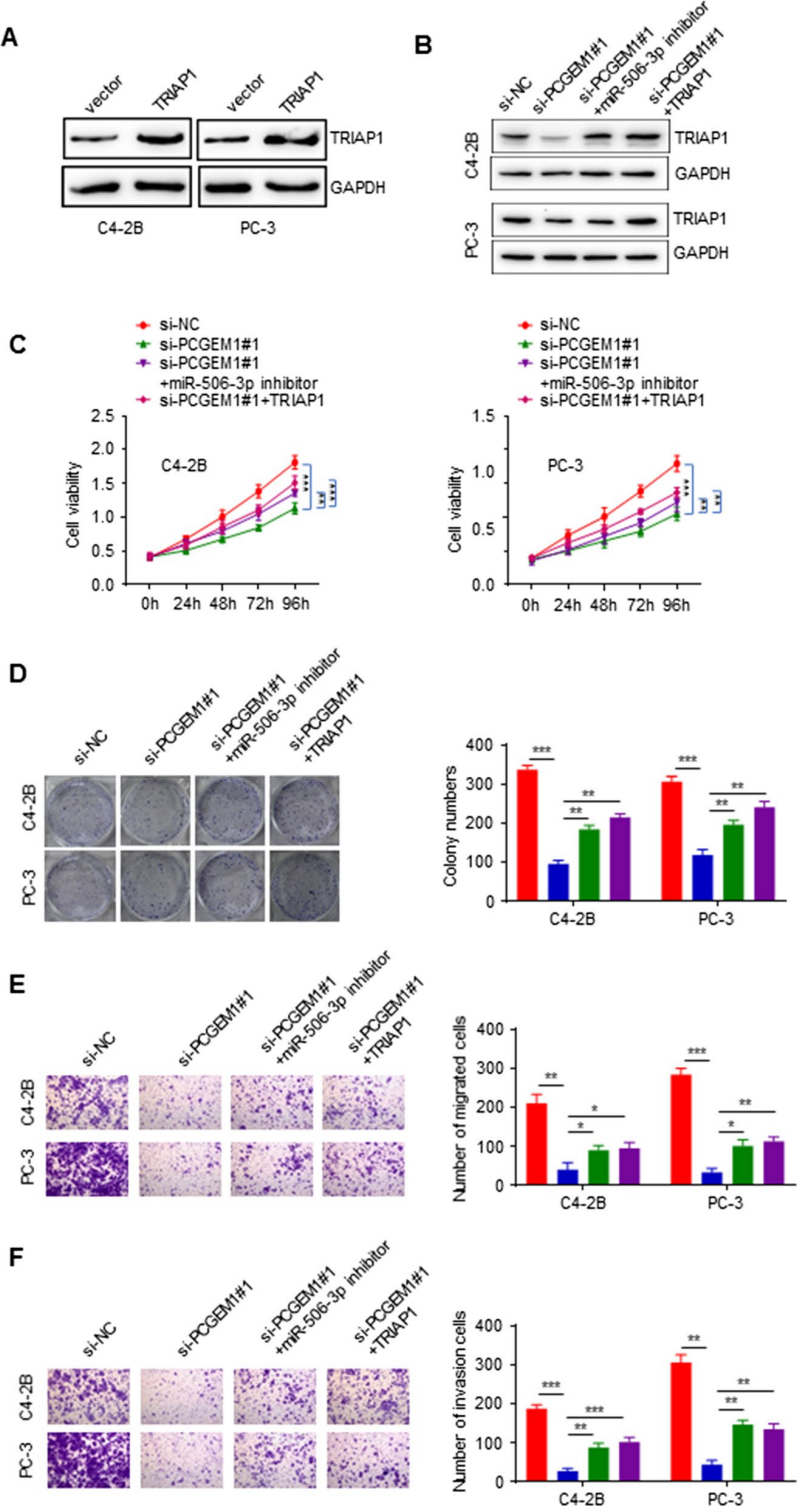
PCGEM1 regulated the biological function of PC-3 and C4-2B cells by modulating miR-506-3p/TRIAP1. **A**, **B** C4-2B and PC-3 cell proliferation was measured through CCK-8 and colony formation assays. **C**, **D** Cell invasion and migration were measured by Transwell assays. *P < 0.05, **P < 0.01, ***P < 0.001

WB were used to measure the expression of PC-3 and C4-2B cells which stably transfected with TRIAP1 overexpression plasmids or control vectors. TRIAP1 overexpression plasmids could effectively overexpression TRIAP1 (Fig. 5A). When cells were transfected with si-PCGEM1#1, the expression of TRIAP1 were inhibited, but the suppression were restored when cotransfection of the miR-506-3p inhibitor or the TRIAP1 overexpression plasmid (Fig. 5B). CCK-8 assay and colony formation assay were performed to determine the impact of PCGEM1 on PCa cell lines through regulating TRIAP1 and miR‑506‑3p. According to these two assays, when si-PCGEM1#1, si-PCGEM1#1 with miR-506-3p inhibitor and si-PCGEM1#1 with TRIAP1 were transfected, the viability and colony formation ability were suppressed compared with si-NC. Cotransfection of miR-506-3p inhibitor or the TRIAP1 overexpression plasmid restored the suppression of si-PCGEM1#1 (Fig. 5C, D). In the Transwell assays, cell invasion and migration were suppressed when cells were transfected with si-PCGEM1#1, si-PCGEM1#1 with miR-506-3p inhibitor and si-PCGEM1#1 with TRIAP1 when compared with si-NC. Cotransfection of the miR-506-3p inhibitor or the TRIAP1 overexpression plasmid restored the suppression of si-PCGEM1#1 (Fig. 5E, F). These results demonstrated that PCGEM1 regulated the biological function of PCa cells by interacting with miR‑506‑3p to upregulate TRIAP1.

### PCGEM1 knockdown inhibited tumor growth in animal models

**Fig. 6:**
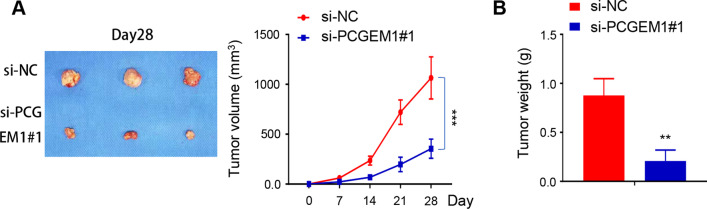
PCGEM1 knockdown inhibited tumor growth in animal models. **A**, **B** The tumors volume and weight in nude mice injected with PC-3 cells transfected with si-NC and si-PCGEM1#1

For further verification the role of PCGEM1 in animal models, PC-3 cells transfected with si-NC and si-PCGEM1#1 were injected into mice subcutaneously, then the tumor volume and weight were measured. We found that the tumor volume and weight in nude mice which injected with PC-3 cells transfected with si-PCGEM1#1 were remarkably reduced, compared with si-NC in nude mice (Fig. 6A, B).

## Discussion

The morbidity and mortality of prostate cancer continue to increase each year, which is a serious threat to men’s health [19]. The formation of prostate cancer is the result of multigene interactions, but the specific mechanism of prostate cancer is not completely clear. In recent years, the important role of lncRNAs in regulating cancer progression has received more attention [20]. LncRNAs are often abnormally expressed in tumors and affect biological processes by regulating miRNA transcription [20]. With the emergence of new research methods, the role of lncRNAs in tumors has been gradually clarified [21]. LncRNA PCGEM1 has been identified as a carcinogenic molecule and has been studied in ovarian cancer, cervical cancer, gastric cancer and prostate cancer. PCGEM1 overexpression might cause carcinogenesis in ovarian cancer and affect its development by modulating YAP, P70S6K, RhoA, Bcl-xL and MMP2 protein levels [22]. PCGEM1 accelerates cell proliferation, invasion and migration by targeting miR-182, while overexpression of PCGEM1 is associated with dismal prognostic outcome, lymph node metastasis and distant metastasis in cervical cancer cases [17]. PCGEM1 promotes gastric cancer metastasis and invasion by regulating SNAI1 [23]. PCGEM1 overexpression has also been detected in PCa and was found to accelerate LNCaP cell proliferation via interaction with miR-145 [5, 24, 25]. Consistent with the results of other studies, in this study, we revealed the role of PCGEM1 in PCa. PCGEM1 was more highly expressed in PCa tissues and cells, high PCGEM1 expression was associated with high Gleason score, distant metastasis and extracapsular extension. and knockdown of PCGEM1 inhibited the proliferation, migration and invasion of PC-3 and C4-2B prostate cancer cells in vitro and in vivo. These findings indicate that PCGEM1 has important biological functions in prostate cancer.

Numerous studies have proposed that lncRNAs can be used as ceRNAs, and lncRNAs can be combined with targeted miRNAs to eliminate endogenous miRNAs and inhibit the transcription of their targeted mRNAs [26]. First, we determined that PCGEM1 mainly plays a role in the cytoplasm, suggesting that it can function as a ceRNA. Second, we performed bioinformatics analysis on the miRNA recognition sequence of PCGEM1 and found that there were more than 20 miRNA binding sites. After a detailed investigation, miR-506-3p was detected. To further confirm the underlying molecular mechanism involved, we performed a luciferase reporter gene experiment to detect the direct binding ability of the full-length transcript of PCGEM1 and miR-506-3p. Biotin-labeled pull-down assay results indicated that PCGEM1 could be pulled down by the miR-506-3p probe but not the NC probe. Furthermore, RIP assay results also confirmed that PCGEM1 and miR-506-3p could be markedly enriched using anti-Ago2 compared to anti-IgG antibodies. Further research demonstrated that PCGEM1 was negatively correlated with the expression of miR-506-3p in prostate cancer tissues, and PCGEM1 directly regulated the expression of miR-506-3p.

MiRNAs are often aberrantly expressed in cancers. They are involved in development and cell differentiation and regulate the cell cycle and physiological processes [27, 28]. MiRNAs regulate tumor progression and metastasis by interacting with target genes [29]. Studies have shown that miR-506-3p inhibits cell proliferation and metastasis, causes apoptosis, arrests the cell cycle, and plays a role as a novel tumor suppressor [18, 30–32]. In our research, the expression of miR-506-3p was low in PCa, consistent with other studies [32]. Simultaneously, we found that miR-506-3p was downstream of PCGEM1. Using bioinformatics analysis, dual-luciferase reporter assays, and correlation analysis in PCa tissues, we demonstrated that miR-506-3p could also interact with the 3′-UTR of TRIAP1. TRIAP1 could bind to HSP70 in the cytoplasm and inhibit the formation of apoptosomes and caspase-9 activation, and had been shown to be upregulated in many types of cancers. TRIAP1 reported to be associated with resistance of apoptosis in different human malignancies [33]. PeCa with high TRIAP1 expression had a high risk of recurrence and poor survival [13]. TRIAP1 also increased the risk of recurrence or metastasis in afflicted patients with non-small cell lung cancer (NSCLC) following irradiation [14]. Knockdown of TRIAP1 inhibited the ability of proliferation, apoptosis, migration and invasion of thyroid cancer, oral squamous cell carcinoma (OSCC) and Lung cancer cells [34–36]. In our study, we revealed that TRIAP1 was highly expressed in PCa tissues, and the expression of TRIAP1 was negatively correlated with the expression of miR-506-3p and positively correlated with the expression of PCGEM1. Overexpression of miR-506-3p inhibited the expression of TRIAP1 protein effectively. Further research showed that cotransfection of a miR-506-3p inhibitor with a TRIAP1 overexpression plasmid restored the suppressive effect of PCGEM1 knockdown on the proliferation, migration and invasion of prostate cancer.

In summary, we demonstrated for the first time that PCGEM1 were upregulationed in prostate cancer and cell lines. PCGEM1 knockdown inhinited the tumor growth in vitro and in vivo. Our study confirmed that PCGEM1 can be used as a biomarker for the early diagnosis of prostate cancer and can also be used as a clinical target to treat prostate cancer.

## Conclusion

According to the findings in this work, PCGEM1 expression is increased in PCa cells and tissues, enhancing PCa proliferation, migration and invasion through sponging of miR-506 to increase TRIAP1 expression. This work highlights the important role of PCGEM1/miR-506-3p/TRIAP1 in PCa. Further elucidating the role of PCGEM1 in PCa could identify novel targets for PCa treatment and provide novel biomarkers for prognosis.

## Supplementary Information

**Additional file 1: Fig. S1**. A: The protein expression levels of PCGEM1 in prostate cancer with different Gleason scores were measured by immunohistochemical staining. B: The protein expression levels of TRIAP1 in prostate cancer with different Gleason scores were measured by immunohistochemical staining.

**Additional file 2: Fig. S2**. A: miR-506-3p has a lower expression level in prostate cancer when compared to normal tissues according to TCGA database. B: TRIAP1has a higher expression level in prostate cancer, especially in metastatic prostate cancer (N1), when compared with normal tissues according to TCGA database. C: TRIAP1 has a higher expression level in prostate cancer, especially in high Gleason score prostate cancer, when compared with normal tissues according to TCGA database.

## Data Availability

The datasets generated/analysed during the current study are available.

## References

[1] Siegel RL, Miller KD, Jemal A (2019). Cancer statistics, 2019. CA Cancer J Clin.

[2] Garnick MB, Fair WR (1998). Combating prostate cancer. Sci Am.

[3] Paraskevopoulou MD, Hatzigeorgiou AG (2016). Analyzing MiRNA–LncRNA interactions. Methods Mol Biol.

[4] Ma Y (2018). Membrane-lipid associated lncRNA: a new regulator in cancer signaling. Cancer Lett.

[5] Srikantan V (2000). PCGEM1, a prostate-specific gene, is overexpressed in prostate cancer. Proc Natl Acad Sci USA.

[6] Petrovics G (2004). Elevated expression of PCGEM1, a prostate-specific gene with cell growth-promoting function, is associated with high-risk prostate cancer patients. Oncogene.

[7] Romanuik TL (2010). LNCaP Atlas: gene expression associated with in vivo progression to castration-recurrent prostate cancer. BMC Med Genom.

[8] Yang L (2013). lncRNA-dependent mechanisms of androgen-receptor-regulated gene activation programs. Nature.

[9] Mendell JT (2005). MicroRNAs: critical regulators of development, cellular physiology and malignancy. Cell Cycle.

[10] Filella X, Foj L (2017). miRNAs as novel biomarkers in the management of prostate cancer. Clin Chem Lab Med.

[11] Liang TS (2019). MicroRNA-506 inhibits tumor growth and metastasis in nasopharyngeal carcinoma through the inactivation of the Wnt/β-catenin signaling pathway by down-regulating LHX2. J Exp Clin Cancer Res.

[12] Liang J, Liu N, Xin H (2019). Knockdown long non-coding RNA PEG10 inhibits proliferation, migration and invasion of glioma cell line U251 by regulating miR-506. Gen Physiol Biophys.

[13] Zhang J (2019). High TRIAP1 expression in penile carcinoma is associated with high risk of recurrence and poor survival. Ann Transl Med.

[14] Hao CC (2020). TRIAP1 knockdown sensitizes non-small cell lung cancer to ionizing radiation by disrupting redox homeostasis. Thorac Cancer.

[15] Cline MS (2013). Exploring TCGA pan-cancer data at the UCSC cancer genomics browser. Sci Rep.

[16] Wang C (2018). The downregulated long noncoding RNA DHRS4-AS1 is protumoral and associated with the prognosis of clear cell renal cell carcinoma. Oncol Targets Ther.

[17] Zhang Q, Zheng J, Liu L (2019). The long noncoding RNA PCGEM1 promotes cell proliferation, migration and invasion via targeting the miR-182/FBXW11 axis in cervical cancer. Cancer Cell Int.

[18] Wang Y (2019). MiR-506-3p suppresses the proliferation of ovarian cancer cells by negatively regulating the expression of MTMR6. J Biosci.

[19] Luo J (2019). LncRNA-p21 alters the antiandrogen enzalutamide-induced prostate cancer neuroendocrine differentiation via modulating the EZH2/STAT3 signaling. Nat Commun.

[20] de Oliveira JC (2019). Long non-coding RNAs in cancer: another layer of complexity. J Gene Med.

[21] Anastasiadou E, Jacob LS, Slack FJ (2018). Non-coding RNA networks in cancer. Nat Rev Cancer.

[22] Chen S (2018). LncRNA PCGEM1 induces ovarian carcinoma tumorigenesis and progression through RhoA pathway. Cell Physiol Biochem.

[23] Zhang J, Jin HY (2019). Hypoxia-induced LncRNA PCGEM1 promotes invasion and metastasis of gastric cancer through regulating SNAI1. Clin Transl Oncol.

[24] He JH (2014). Reciprocal regulation of PCGEM1 and miR-145 promote proliferation of LNCaP prostate cancer cells. J Exp Clin Cancer Res.

[25] Ho TT (2016). Regulation of PCGEM1 by p54/nrb in prostate cancer. Sci Rep.

[26] Salmena L (2011). A ceRNA hypothesis: the Rosetta Stone of a hidden RNA language?. Cell.

[27] Tutar Y (2012). Pseudogenes. Comp Funct Genom.

[28] Bartel DP (2009). MicroRNAs: target recognition and regulatory functions. Cell.

[29] Tutar Y (2014). miRNA and cancer; computational and experimental approaches. Curr Pharm Biotechnol.

[30] Wu L, Chen Z, Xing Y. MiR-506–3p inhibits cell proliferation, induces cell cycle arrest and apoptosis in retinoblastoma by directly targeting NEK6. Cell Biol Int. 2018. 10.1002/cbin.11041 30080301

[31] Jiashi W (2018). MicroRNA-506-3p inhibits osteosarcoma cell proliferation and metastasis by suppressing RAB3D expression. Aging (Albany NY).

[32] Hu CY (2019). MiR-506-3p acts as a novel tumor suppressor in prostate cancer through targeting GALNT4. Eur Rev Med Pharmacol Sci.

[33] Adams C (2015). Apoptosis inhibitor TRIAP1 is a novel effector of drug resistance. Oncol Rep.

[34] Yu T (2020). Upregulation of TRIAP1 by the lncRNA MFI2-AS1/miR-125a-5p axis promotes thyroid cancer tumorigenesis. Oncol Targets Ther.

[35] Na C (2019). miR-107 targets TRIAP1 to regulate oral squamous cell carcinoma proliferation and migration. Int J Clin Exp Pathol.

[36] Cai P (2020). MicroRNA-107 may regulate lung cancer cell proliferation and apoptosis by targeting TP53 regulated inhibitor of apoptosis 1. Oncol Lett.

