# Supplementary figures and images for: PCGEM1 promotes proliferation, migration and invasion in prostate cancer by sponging miR-506 to upregulate TRIAP1
Source: BMC Urol. 2022 Feb 2;22:14. doi: 10.1186/s12894-022-00969-x (PMC8808993; doi:10.1186/s12894-022-00969-x)

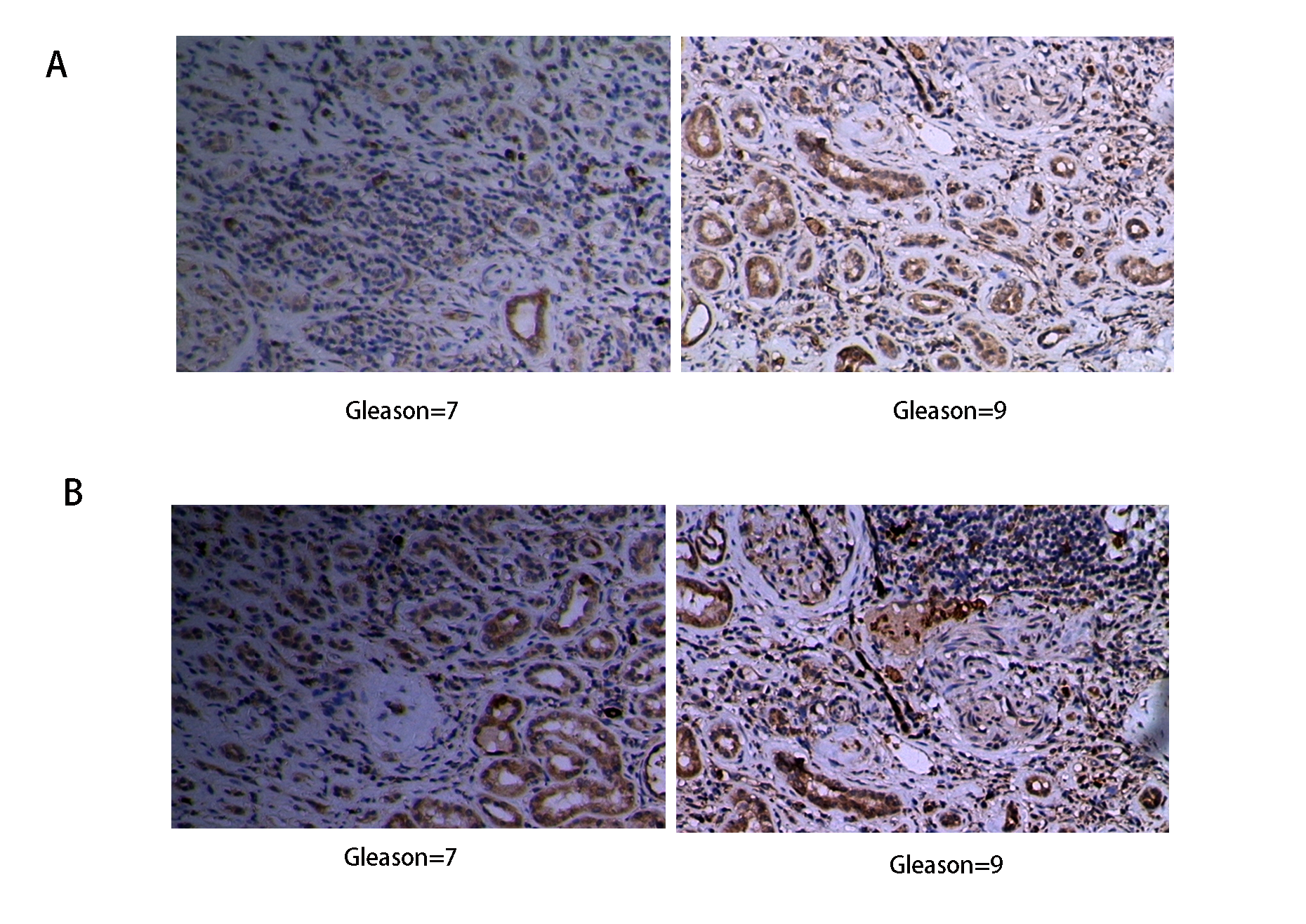

Supplement: Supplementary file 1 — Additional file 1: Fig. S1. A: The protein expression levels of PCGEM1 in prostate cancer with different Gleason scores were measured by immunohistochemical staining. B: The protein expression levels of TRIAP1 in prostate cancer with different Gleason scores were measured by immunohistochemical staining. [file 12894_2022_969_MOESM1_ESM.tif]

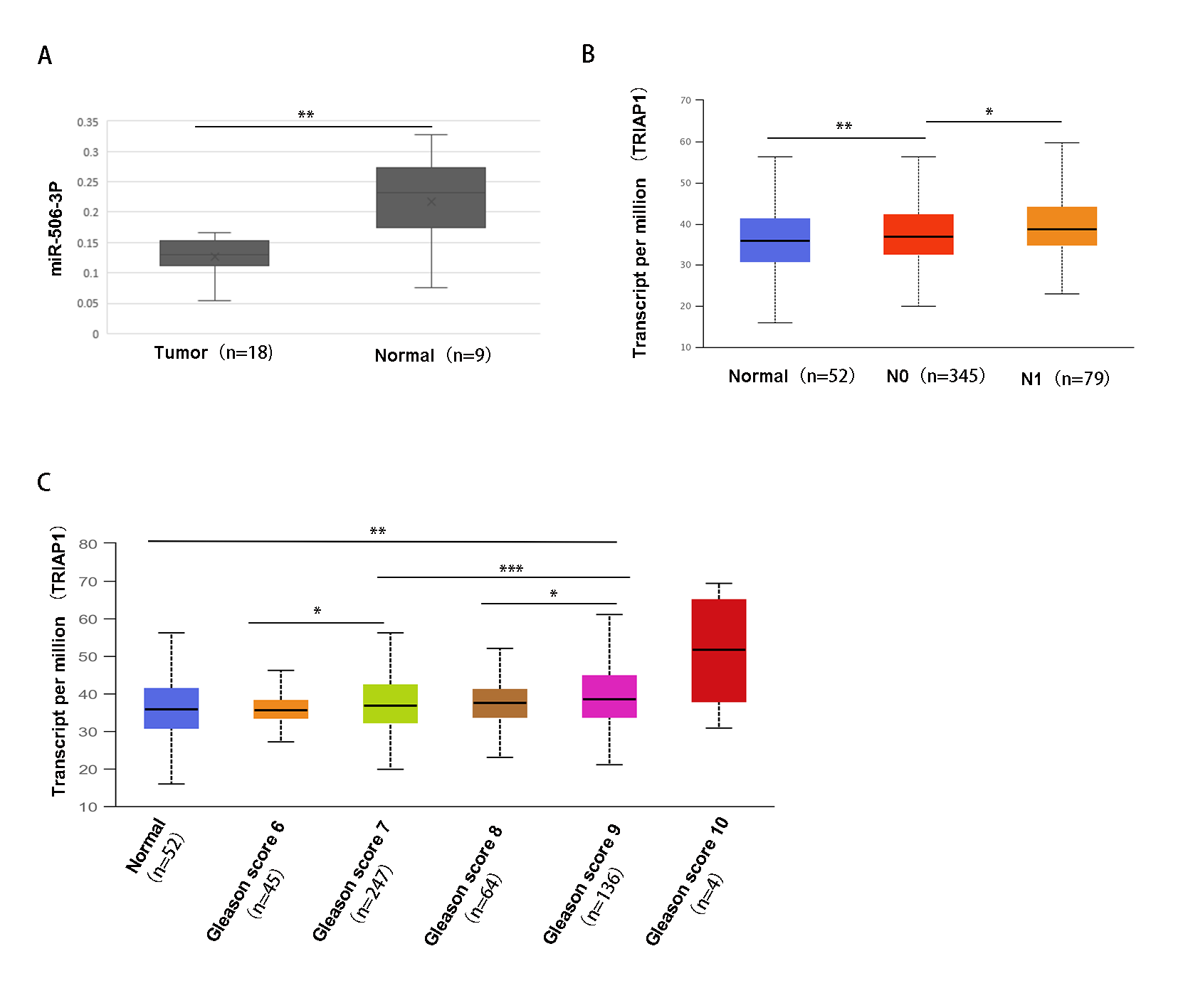

Supplement: Supplementary file 2 — Additional file 2: Fig. S2. A: miR-506-3p has a lower expression level in prostate cancer when compared to normal tissues according to TCGA database. B: TRIAP1has a higher expression level in prostate cancer, especially in metastatic prostate cancer (N1), when compared with normal tissues according to TCGA database. C: TRIAP1 has a higher expression level in prostate cancer, especially in high Gleason score prostate cancer, when compared with normal tissues according to TCGA database. [file 12894_2022_969_MOESM2_ESM.tif]
